# Physical activity as a molecular modulator to enhance immunotherapy and drug sensitivity in the endometrial cancer–comorbidity continuum

**DOI:** 10.3389/fonc.2026.1791875

**Published:** 2026-07-07

**Authors:** Jing Wang, Zhenyu Yang, Yang Wu, Menghao Tang, Yihan Wang, Hongyun Zheng, Xinming Ye, Mei Du

**Affiliations:** School of Sports Science and Engineering, East China University of Science and Technology, Shanghai, China

**Keywords:** disease prevention, drug sensitivity, endometrial cancer, hypertension, kidney dysfunction, physical activity

## Abstract

Effective treatment of endometrial cancer (EC) is frequently challenged by therapeutic resistance, immunosuppressive tumor microenvironments (TME), and patient comorbidities such as hypertension, kidney dysfunction, and metabolic syndrome. While molecular targeted therapies and immunotherapies (e.g., PD-1 blockade) have emerged as transformative approaches, variable drug sensitivity—often exacerbated by the systemic inflammatory and uremic burden of comorbidities—remains a significant hurdle. Building on the integration of molecular oncology and clinical translation, this review examines physical activity not merely as a preventive measure, but as a systems-level molecular modulator capable of enhancing drug sensitivity and immunotherapy efficacy. We synthesize evidence distinguishing preclinical findings from clinical reality, demonstrating that exercise remodels the TME by repolarizing Tumor-Associated Macrophages (TAMs), reducing Myeloid-Derived Suppressor Cells (MDSCs), and enhancing cytotoxic T cell infiltration. Furthermore, we discuss how exercise-induced shear stress promotes eNOS phosphorylation to normalize tumor vasculature and improve renal perfusion, thereby enhancing drug delivery and mitigating nephrotoxicity. We propose an integrated “EC-Comorbidity Continuum” perspective where personalized exercise prescriptions—distinguishing between acute perfusion benefits and chronic immune priming—serve as a non-pharmacological adjuvant to optimize molecular responses and overcome multidrug resistance.

## 1 Introduction

**Figure 1:**
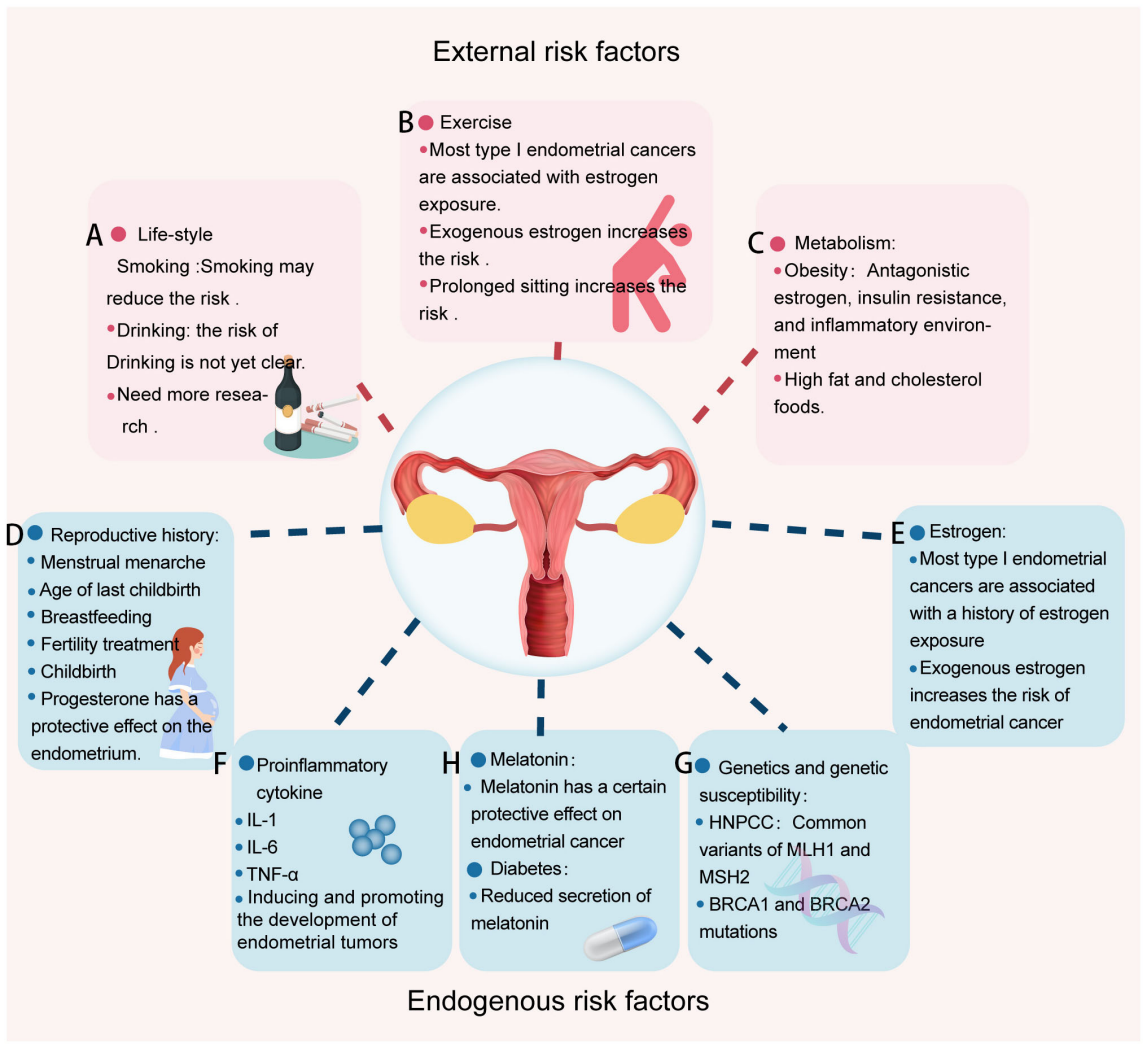
External risk factors and endogenous risk factors. **(A)** Life-style: Smoking may reduce the risk of endometrial cancer, while the association between alcohol consumption and endometrial cancer risk remains unclear and requires further investigation. **(B)** Exercise: Most type I endometrial cancers are associated with estrogen exposure, with exogenous estrogen and prolonged sitting increasing the risk, whereas physical activity is a key modifiable factor. **(C)** Metabolismz: Obesity contributes to endometrial cancer risk through antagonistic estrogen effects, insulin resistance, and a pro-inflammatory environment, with high-fat and high-cholesterol diets further exacerbating this risk. **(D)** Reproductive history: Menstrual menarche, age at last childbirth, breastfeeding, fertility treatment, and childbirth influence endometrial cancer risk, while progesterone exerts a protective effect on the endometrium. **(E)** Estrogen: Most type I endometrial cancers are associated with a history of estrogen exposure, and exogenous estrogen increases the risk of endometrial cancer. **(F)** Proinflammatory cytokine: Proinflammatory cytokines, including IL-1, IL-6, and TNF-α, induce and promote the development of endometrial tumors. **(G)** Genetics and genetic susceptibility: Genetic susceptibility to endometrial cancer includes hereditary nonpolyposis colorectal cancer associated with common variants of MLH1 and MSH2, as well as BRCA1 and BRCA2 mutations. **(H)** Melatonin: Melatonin has a certain protective effect on endometrial cancer, and metabolic disorders like diabetes can reduce its secretion.

Endometrial cancer (EC) is the most common gynecologic malignancy in developed countries, with a steadily rising incidence that parallels global increases in obesity and metabolic disorders (1). The disease predominantly affects postmenopausal women, and its pathogenesis is closely linked to a metabolic-hormonal axis driven by obesity, type 2 diabetes mellitus (T2DM), and physical inactivity (2). These conditions fuel the insulin/IGF-1 signaling pathway, promoting tumor cell proliferation, and underlie a broader spectrum of cardiometabolic comorbidities, notably hypertension and chronic kidney disease (CKD), that frequently coexist in this population (**Figure 1**) (3–5). Contemporary molecular classification of EC into POLE-ultramutated, mismatch repair-deficient (MMRd), no specific molecular profile (NSMP), and p53-abnormal subtypes has further refined our understanding of its biology, revealing distinct tumor microenvironments (TME) and differential immunogenicity that are critical when considering therapeutic strategies.

While a substantial body of epidemiological evidence establishes physical activity (PA) as a protective factor in EC risk reduction and prevention (6), and as a cornerstone in managing hypertension and CKD (7), the clinical challenge in this review extends beyond prevention. We focus specifically on the treatment context: patients with active, molecularly heterogeneous EC who present with a complex multimorbidity profile and are receiving systemic therapies (8). In this setting, comorbidities such as hypertension and CKD are not merely co-existing conditions but constitute active biological barriers to effective treatment. The systemic inflammatory burden and accumulation of uremic toxins associated with CKD can accelerate T-cell exhaustion, directly blunting the efficacy of emerging immunotherapies (e.g., PD-1/PD-L1 inhibitors) (9–11). Concurrently, the endothelial dysfunction and chaotic, leaky tumor vasculature—often exacerbated by hypertension—impede effective delivery of chemotherapeutic agents and immunoglobulins (12, 13).

This review synthesizes current epidemiological, experimental, and clinical evidence, explicitly differentiating between preventive and treatment-sensitizing roles, to examine how PA influences EC, hypertension, and kidney dysfunction through convergent biological mechanisms. We propose a hypothesis-generating framework: PA as a systems-level molecular modulator capable of dismantling these comorbidity-driven therapeutic barriers. Crucially, we distinguish preclinical findings from clinical reality, and EC-specific evidence from data extrapolated from other solid tumors. We emphasize a critical analysis of the evidence, highlighting where mechanisms are well-established versus where they remain hypothetical. By framing PA as a targeted molecular intervention, this review aims to provide a mechanistic insight and a balanced clinical perspective to inform future research and support the considered, evidence-guided incorporation of exercise into comprehensive management strategies for this high-need patient population.

## 2 Physical activity in disease prevention: a brief background

### 2.1 Epidemiological evidence supporting exercise in disease prevention

Extensive epidemiological studies provide compelling evidence that physical activity plays a crucial role in the prevention of chronic diseases such as endometrial cancer, hypertension, and kidney dysfunction. These studies consistently demonstrate that higher levels of physical activity are associated with a reduced risk of these diseases, reinforcing the importance of exercise as a preventive measure. For endometrial cancer, research has consistently shown that women who engage in regular physical activity have a significantly lower risk of developing the disease. A meta-analysis of cohort studies reported that women who engaged in regular physical activity exhibited a substantially lower risk of endometrial cancer compared with those who were sedentary (14, 15). Exercise has been shown to influence several biological mechanisms, including the reduction of body fat, improved insulin sensitivity, and regulation of estrogen levels (16). These factors are well-established contributors to cancer development, and exercise plays a critical role in modulating them. In hypertension, exercise has long been recognized as an effective intervention for both prevention and management. Studies consistently demonstrate that physical activity, particularly aerobic exercise, significantly reduces both systolic and diastolic blood pressure. Exercise improves endothelial function, increases arterial compliance, and reduces vascular resistance, all of which contribute to better blood pressure regulation (17). For kidney dysfunction, the association between physical activity and improved kidney health is well-supported by epidemiological evidence. Studies have shown that individuals who engage in regular physical activity have better renal hemodynamics, slower declines in glomerular filtration rate, and reduced levels of proteinuria (18). Exercise has been linked to improved kidney function and a decreased risk of developing CKD, especially in populations with comorbidities such as hypertension and diabetes (19). Regular physical activity reduces inflammation and improves metabolic health, both of which are crucial in preventing CKD progression.

### 2.2 Exercise as a preventive baseline

Given the interconnected nature of endometrial cancer, hypertension, and kidney dysfunction, exercise serves as an effective preventive and therapeutic strategy for managing these diseases. Regular physical activity addresses common risk factors such as insulin resistance, obesity, and inflammation, which contribute to the development and progression of these conditions (20). Exercise not only helps to prevent the onset of these diseases but also offers therapeutic benefits for individuals already affected. In clinical practice, exercise should be integrated into treatment plans for patients with hypertension, kidney disease, and a history of endometrial cancer. Exercise interventions can be tailored to the individual’s health status and fitness level, with specific recommendations for aerobic and resistance training to optimize health outcomes. Personalized exercise prescriptions, combined with lifestyle modifications and pharmacological treatments, provide a holistic approach to disease management.

## 3 Physical activity as a treatment sensitizer in active endometrial cancer

### 3.1 Exercise-induced metabolic and inflammatory reprogramming in endometrial cancer: dissecting mechanism from evidence

Endometrial cancer development is strongly influenced by prolonged estrogen exposure in conjunction with metabolic dysfunction and chronic low-grade inflammation (**Figure 1**) (21). Crucially, the biological context is shaped not simply by body mass index (BMI) but by specific body composition parameters—visceral adiposity, myosteatosis, and sarcopenic obesity—which are more informative in shaping the TME and determining metabolic risk (22). These metrics serve as critical integrators, linking metabolic and inflammatory pathways to EC biology. PAs ability to reduce endometrial cancer risk and potentially modulate the TME is mediated through the coordinated regulation of these interconnected processes. A central hypothesis, supported by preclinical and indirect clinical evidence, involves exercise-mediated improvements in insulin sensitivity and reductions in specific adipose depots, which collectively lower circulating insulin and insulin-like growth factor-1 (IGF-1) signaling. Given the mitogenic and antiapoptotic roles of the insulin/IGF axis in endometrial tissue (23), attenuation of this pathway represents a key route through which exercise constrains tumor-promoting signals. Reduction of visceral adiposity further contributes by limiting peripheral estrogen synthesis, as adipose tissue serves as a primary site of aromatization in postmenopausal women. By decreasing visceral fat mass and preserving skeletal muscle quality and quantity, PAs lowers estrogen bioavailability to the endometrium and mitigates hormonally driven proliferation (**Figure 1**). Importantly, during active cancer therapy, myosteatosis and sarcopenia are not merely metabolic risk factors but are directly associated with altered pharmacokinetics of chemotherapeutic agents and increased dose-limiting toxicities. The reduction of visceral fat and preservation of skeletal muscle quality through exercise is therefore hypothesized to improve treatment tolerance and overall prognosis in active EC patients undergoing systemic therapies (22).

**Figure 2:**
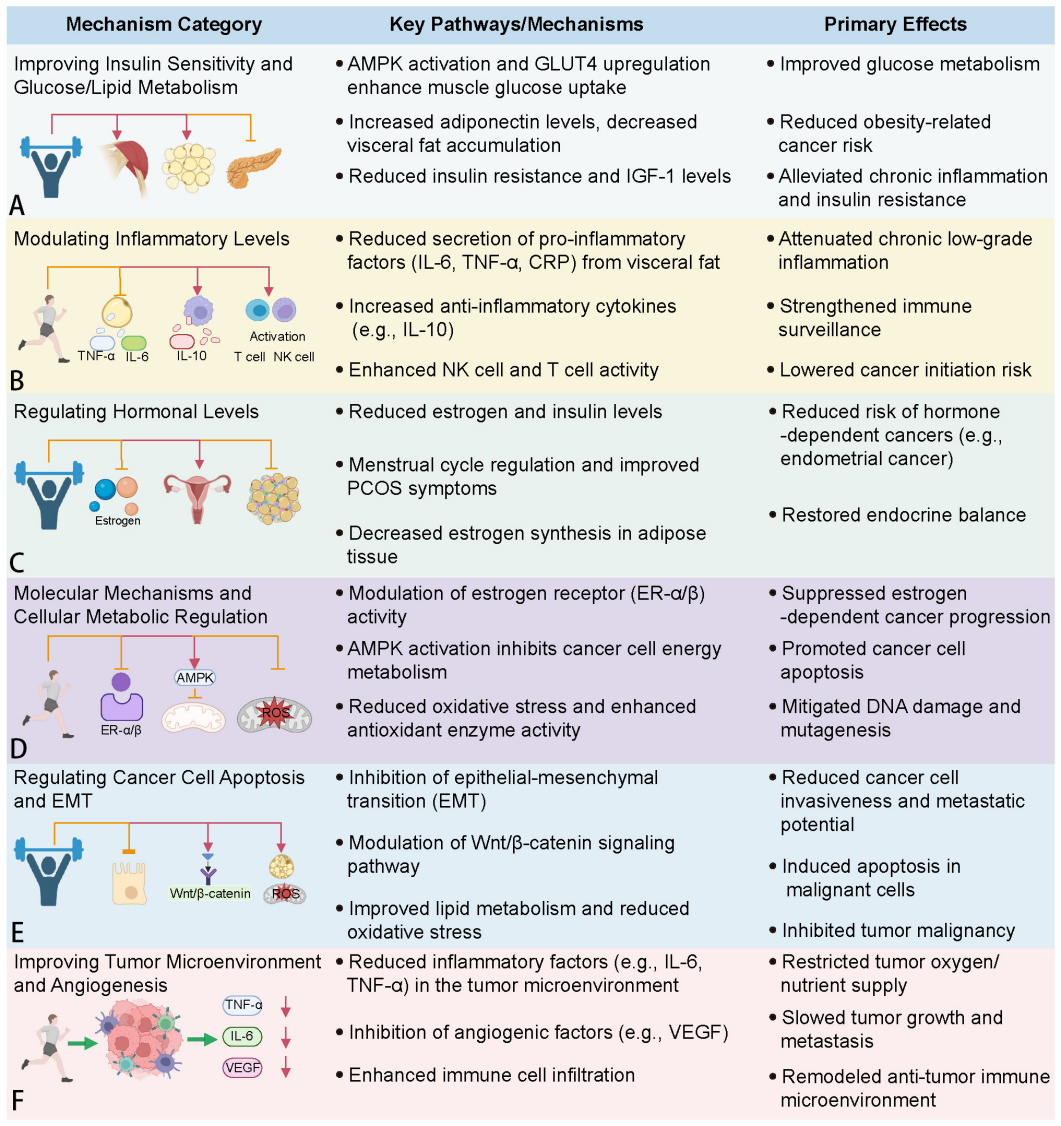
Exercise-mediated mechanistic pathways in cancer prevention and progression. **(A)** Enhancing Insulin Sensitivity and Glucose and Lipid Metabolic Regulation: Exercise activates AMPK and upregulates GLUT4, increases adiponectin levels, reduces visceral fat accumulation, and lowers insulin resistance and IGF-1 levels, leading to improved glucose metabolism, reduced obesity-related cancer risk, and alleviated chronic inflammation. **(B)** Modulating Inflammatory Levels: Exercise reduces the secretion of pro-inflammatory factors such as IL-6, TNF-α, and CRP from visceral fat, increases anti-inflammatory cytokines like IL-10, enhances NK cell and T-cell activity, and thereby attenuates chronic low-grade inflammation while strengthening immune surveillance. **(C)** Regulating Hormonal Levels: Exercise lowers estrogen and insulin levels, improves menstrual cycle regulation and PCOS-related symptoms, and decreases estrogen synthesis in adipose tissue, resulting in restored endocrine balance and a reduced risk of hormone-dependent cancers, including endometrial cancer. **(D)** Molecular Mechanisms and Cellular Metabolic Regulation: Exercise modulates estrogen receptor (ER-α/β) activity, activates AMPK to inhibit cancer cell energy metabolism, and reduces oxidative stress while enhancing antioxidant enzyme activity, thereby suppressing estrogen-dependent cancer progression and mitigating DNA damage and mutagenesis. **(E)** Regulating Cancer Cell Apoptosis and EMT: Exercise inhibits EMT, modulates the Wnt/β-catenin signaling pathway, and improves lipid metabolism while reducing oxidative stress, leading to decreased cancer cell invasiveness, induced apoptosis in malignant cells, and inhibited tumor malignancy. **(F)** Improving Tumor Microenvironment and Angiogenesis: Exercise reduces inflammatory factors such as IL-6 and TNF-α within the tumor microenvironment, inhibits angiogenic factors including VEGF, and enhances immune cell infiltration, thereby restricting tumor oxygen and nutrient supply, slowing tumor growth and metastasis, and remodeling the anti-tumor immune microenvironment.

Mechanistically, preclinical models consistently demonstrate that exercise induces metabolic reprogramming through activation of AMPK, a central energy sensor that suppresses anabolic pathways including the PI3K/AKT/mTOR signaling axis—a pathway frequently hyperactivated in type I EC (24, 25). While direct AMPK-mediated tumor suppression is robust in rodent studies, human clinical evidence remains indirect, primarily supporting systemic adaptations like improved glycemic control and reduced circulating insulin/IGF-1 levels via GLUT4 upregulation in skeletal muscle (26). These systemic changes may indirectly downregulate tumoral NF-κB signaling, limiting oxidative stress and mutagenic pressure within endometrial tissue (**Figure 2**) (27–29). The impact of these systemic adaptations likely varies across the molecular subtypes of EC. For instance, in the context of POLEmut and MMRd tumors with high neoantigen loads, reducing systemic inflammation might be less critical for immune recognition than in NSMP or p53-abnormal tumors, where an inflamed TME must be actively established. Genetic susceptibility further shapes baseline risk and may influence interindividual variability in the magnitude of exercise-related immunometabolic modulation. Lynch syndrome (MMRd) and BRCA1/2 mutations confer substantially increased lifetime risk of EC (30, 31), but the degree to which these inherited factors influence exercise responsiveness remains an important, unanswered question. While AMPK-mediated tumor suppression is robust in rodent studies, human clinical evidence primarily supports systemic adaptations, such as improved glycemic control and reduced circulating insulin/IGF-1 levels via GLUT4 upregulation in skeletal muscle (26). These systemic changes indirectly downregulate tumoral NF-κB signaling, thereby limiting oxidative stress and mutagenic pressure within the endometrial tissue (**Figure 2**) (27, 28). Genetic susceptibility further shapes baseline risk and may influence interindividual variability in the magnitude of exercise related risk reduction. Lynch syndrome, associated with pathogenic variants in DNA mismatch repair genes including MLH1 and MSH2, confers a substantially increased lifetime risk of endometrial cancer (30). Mutations in BRCA1 and BRCA2 have also been linked to elevated risk in selected populations (**Figure 1**) (31). These inherited factors may amplify the effects of hormonal imbalance, metabolic dysfunction, and inflammation, but do not negate the capacity of exercise mediated pathway modulation to favorably influence disease risk.

### 3.2 Endocrine and immunometabolic regulation of endometrial cancer risk through exercise

Beyond metabolic regulation, exercise exerts profound effects on endocrine and immunometabolic pathways that collectively shape endometrial cancer susceptibility. Physical activity lowers peripheral estrogen production not only by reducing visceral adiposity but also by modulating aromatase expression within inflamed adipose tissue. Exercise reduces local levels of Prostaglandin E2 (PGE2) and TNF-α, which are potent inducers of the CYP19A1 (aromatase) gene, thereby attenuating local estrogen synthesis even in the absence of significant weight loss (32). Exercise may also improve the progesterone to estrogen balance, which limits endometrial hyperplasia and malignant transformation. In premenopausal women, exercise has been associated with improved menstrual cycle regularity and attenuation of polycystic ovary syndrome related endocrine abnormalities, accompanied by reductions in estrogen and insulin levels and suppression of adipose derived estrogen synthesis (**Figure 2**) (33). Exercise induced enhancement of insulin sensitivity further downregulates the insulin and IGF axis, resulting in lower circulating insulin and IGF levels, reduced mitogenic signaling, and reinforcement of tumor suppressive activity (34). Favorable shifts in adipokine profiles strengthen this protective endocrine milieu, with decreased leptin and increased adiponectin supporting improved metabolic control and reduced inflammatory signaling (35). Within this hormonal framework, reproductive factors determine cumulative exposure to unopposed estrogen across the life course. Earlier menarche, nulliparity, and infertility related conditions, including certain fertility treatments, are associated with prolonged estrogen stimulation, whereas progesterone counteracts estrogen driven endometrial proliferation (3). Consistently, pregnancy, breastfeeding, and progestin containing contraceptives increase progesterone exposure and are linked to reduced endometrial cancer risk. Exercise may complement these protective influences by sustaining long term metabolic and endocrine homeostasis, thereby modulating the biological context in which reproductive factors exert their effects (**Figure 1**) (36).

Chronic low grade inflammation represents a critical mediator linking metabolic and hormonal dysregulation to tumor initiation and progression in endometrial cancer (**Figure 1**) (37). Regular physical activity reduces systemic inflammatory burden by lowering markers such as C-reactive protein, TNF-α, and IL-6, while promoting an anti-inflammatory cytokine profile characterized by increased IL-10 levels (**Figure 2**) (38). These changes not only suppress pro tumorigenic signaling but also enhance immune surveillance. Exercise has been shown to improve the function of immune effector cells, including natural killer cells and cytotoxic T lymphocytes, while concurrently remodeling the myeloid compartment (**Figure 2**). Specifically, physical activity promotes the repolarization of Tumor-Associated Macrophages (TAMs) from a pro-tumor M2-like phenotype to an anti-tumor M1-like phenotype and facilitates the depletion of Myeloid-Derived Suppressor Cells (MDSCs), thereby overcoming the immunosuppressive barrier (39, 40). Furthermore, it is crucial to consider these immunomodulatory effects within the context of the four molecular subtypes of EC. MMRd and POLEmut tumors are inherently immunogenic, characterized by high T-cell infiltration and a high mutational burden, and are thus most responsive to immune checkpoint inhibitors (ICIs). In this context, PAs primary role may shift from one of immune priming to one of supporting vascular delivery and metabolic health. Conversely, we hypothesize that exercise-mediated immune modulation—specifically the repolarization of TAMs from an M2 to an M1 phenotype and the functional reinvigoration of exhausted T cells—acts as a critical immune-priming mechanism specifically for NSMP and certain p53-abnormal subtypes (39–41). By reducing systemic inflammatory burden and promoting immune effector cell function (**Figure 2**) (38), exercise may theoretically mechanically shift the TME from an intrinsically immunologically ‘cold’ state to a more inflamed, ‘hot’ phenotype (42), overcoming the specific immune evasion barriers characteristic of these subtypes and creating a permissive environment for subsequent checkpoint blockade. This is a key, hypothesis-generating proposition of this review that requires prospective validation in EC-specific trials, as evidence for this mechanism is currently extrapolated from preclinical and pan-cancer clinical studies. Emerging evidence suggests that circadian related endocrine signals intersect with metabolic and inflammatory pathways relevant to endometrial carcinogenesis. Melatonin, a circadian regulated hormone with anti-inflammatory and antioxidant properties, may modulate estrogen signaling. Metabolic disorders such as diabetes are associated with reduced melatonin secretion, potentially increasing susceptibility to endometrial cancer (**Figure 1**) (43). By stabilizing circadian rhythms, regular physical activity may indirectly support melatonin homeostasis, providing an additional mechanism through which exercise reinforces inflammatory control and reduces carcinogenic risk (44).

### 3.3 Exercise-mediated modulation of the tumor microenvironment

In addition to systemic metabolic, endocrine, and immune effects, exercise influences endometrial cancer progression through modulation of the tumor microenvironment (TME). The TME integrates signals from immune cells, vasculature, stromal components, and extracellular matrix, and plays a pivotal role in determining tumor growth and therapeutic responsiveness. Physical activity can attenuate pro inflammatory mediators within the tumor milieu, including IL-6 and TNF-α, while promoting immune cell infiltration, thereby supporting a more effective antitumor immune landscape (**Figure 2**) (45). Crucially, evidence primarily from preclinical models and other solid tumors suggests that exercise promotes tumor vascular normalization, a mechanism of profound relevance to our multimorbid EC population. We are not proposing simple angiogenesis inhibition, but a normalization process. Extrapolating from preclinical models of other solid tumors, exercise-induced laminar shear stress is hypothesized to stimulate endothelial nitric oxide synthase (eNOS) production in endothelial cells (46), improving endothelial function (47) and reducing vascular leakiness. This mechanism is hypothesized to directly counteract the endothelial dysfunction induced by the hypertensive state, thereby repairing chaotic tumor vessels. Simultaneously, this shares a pathway with the hemodynamic improvements seen in the renal cortex, where exercise enhances cardiac output and renal perfusion (48), creating a synergistic axis where PA concurrently improves drug delivery to the tumor and aids clearance of nephrotoxic agents. This ‘vascular bridge’ concept—enhanced delivery of chemotherapeutics and immunoglobulins—remains an inference from pan-cancer models and must be explicitly studied in the EC setting (**Figure 2**) (49). The impact of these changes on the ECM and stromal components represents another layer of hypothesized benefit awaiting direct evidence in EC (**Figure 2**) (50, 51).

## 4 The EC-comorbidity continuum: a hypothesis-generating model for overcoming therapeutic resistance

### 4.1 Interconnections between endometrial cancer, hypertension, and kidney dysfunction: a conceptual model

Endometrial cancer, hypertension, and kidney dysfunction share common pathophysiological mechanisms that justify their grouping in a clinical continuum (52, 53). We argue these three are not an arbitrary selection but are linked by a critically reinforcing triad of insulin resistance, systemic inflammation, and endothelial dysfunction (54–56). The uremic milieu characteristic of CKD actively generates therapeutic resistance through specific, actionable mechanisms. The accumulation of uremic toxins not only impairs adiponectin signaling but also causes direct T-cell toxicity and promotes a state of chronic immune exhaustion characterized by upregulation of inhibitory receptors like PD-1 (9–11, 57, 58). This creates an ‘inflammatory shield’ that directly counteracts the mechanism of action of immunotherapies. Concurrently, hypertension-driven endothelial dysfunction exacerbates the already abnormal, leaky tumor vasculature, creating a physical barrier to drug perfusion and creating hypoxic regions resistant to radiotherapy and chemotherapy (12). Therefore, these comorbidities are critically positioned as direct biological antagonists to both immunotherapy and chemotherapy.

### 4.2 Synergistic effects of exercise: a dual-mechanism model for treatment sensitization

Extrapolating from preclinical data, our central hypothesis posits a hypothesis-generating dual-mechanism model through which PA may hypothetically act as a molecular sensitizer by dismantling these very barriers. First, by reducing systemic inflammatory biomarkers and uremic toxins through improved metabolic function and renal perfusion (19, 59–64), exercise may relieve the chronic inflammatory burden that drives T-cell exhaustion, potentially “making space” for immune reinvigoration (41). Second, by enhancing systemic endothelial function, exercise is hypothesized to promote tumor vascular normalization, creating the aforementioned “vascular bridge” for enhanced drug and immunoglobulin delivery (13). This model is inherently synergistic, uniquely addressing both the pharmacodynamic (immune sensitivity) and pharmacokinetic (drug delivery) obstacles erected by the hypertensive-uremic state in patients with active EC. This distinction between physiological and immunological barriers allows for a nuanced understanding of exercise’s potential, but it is essential to state that clinical validation of this dual-mechanism model within EC cohorts remains completely lacking.

## 5 Clinical applications, limitations, and future directions

### 5.1 Clinical applications of exercise in disease prevention and management

In clinical settings, exercise is increasingly integrated into disease prevention and management strategies for endometrial cancer, hypertension, and kidney dysfunction. The clinical evidence supports the use of exercise as an adjunct to traditional treatments, improving patient outcomes and enhancing quality of life (65). For endometrial cancer, exercise is recognized as an important component of cancer treatment and survivorship care. Clinical trials have shown that exercise helps reduce fatigue, improve physical function, and enhance quality of life in cancer patients undergoing treatment. It also mitigates the side effects of treatment, such as muscle wasting and weight gain, which are common during chemotherapy and radiation (66). Furthermore, exercise has been shown to enhance immune function and improve cardiovascular fitness, contributing to better overall survival rates and reduced cancer recurrence in some studies (67). In hypertension management, exercise is a cornerstone of treatment guidelines. The American College of Cardiology and the American Heart Association recommend at least 150 minutes of moderate-intensity aerobic activity per week as a primary intervention for lowering blood pressure (68). Exercise not only reduces blood pressure but also improves heart health by enhancing endothelial function and reducing arterial stiffness. Resistance training, when combined with aerobic exercise, has been shown to further improve blood pressure control and overall cardiovascular health (69). For kidney dysfunction, clinical trials have demonstrated that exercise improves renal function, enhances cardiovascular health, and reduces the risk of complications associated with CKD. Patients with CKD who engage in regular physical activity experience improved physical performance, better management of metabolic risk factors, and reduced inflammation (70). Exercise also helps maintain muscle strength, which is often compromised in CKD patients due to muscle wasting (71). Furthermore, exercise can slow the progression of kidney disease and delay the need for dialysis in patients with advanced CKD (72).

### 5.2 Personalized exercise prescriptions for disease management

Personalized exercise prescriptions are crucial for optimizing the benefits of physical activity, particularly in patients with chronic diseases such as endometrial cancer, hypertension, and kidney dysfunction. Tailoring exercise interventions based on individual health conditions, fitness levels, and comorbidities ensures that the prescribed exercise is both safe and effective.

For endometrial cancer patients, exercise programs should focus on maintaining a healthy body weight, improving cardiovascular fitness, and enhancing strength and flexibility (73). The intensity and duration of exercise should be adjusted based on the patient’s current physical capacity and treatment status. For example, patients undergoing chemotherapy may begin with low-impact activities such as walking or cycling, gradually increasing intensity as tolerated (74).

In hypertension, the primary focus should be on aerobic exercise. Studies consistently demonstrate that moderate-intensity aerobic activity (e.g., 150 minutes per week) significantly reduces both systolic and diastolic blood pressure (75, 76), aligning with major guideline recommendations (68). For individuals with more severe hypertension, or those with additional risk factors, the exercise prescription should be adjusted to accommodate their cardiovascular status. Resistance training should be integrated, particularly to improve muscle strength, glucose metabolism, and insulin sensitivity (77–79), which are critical for long-term blood pressure control (80).

For kidney disease, exercise prescriptions should be individualized based on the patient’s stage of CKD and overall health. Meta-analyses suggest that combining aerobic and resistance training yields the most significant improvements in renal function for CKD patients (81). In early stages of CKD, moderate-intensity aerobic exercise, such as brisk walking, is recommended to improve cardiovascular function and prevent further renal damage. In more advanced stages, the focus should be on light resistance training and flexibility exercises to prevent muscle wasting and maintain physical function (82).

### 5.3 Integrating exercise into multidisciplinary care

The integration of exercise into multidisciplinary care models is essential for optimizing disease management. Exercise should be viewed as a vital component of a comprehensive treatment plan, alongside pharmacological interventions, dietary modifications, and other lifestyle changes. Healthcare providers should collaborate with exercise specialists and physical therapists to design tailored exercise programs for patients, ensuring they receive the full benefits of physical activity. Recent research underscores the importance of behavioral interventions to enhance patient adherence to exercise programs. Strategies such as motivational interviewing, goal setting, and providing social support can significantly improve exercise adherence (83). Additionally, ongoing monitoring and adjustments to exercise intensity, duration, and type are essential to ensure that the intervention remains effective and safe (84). Emerging clinical and translational research continues to explore the molecular mechanisms of exercise in chronic disease management (85). Studies are investigating how exercise affects inflammatory pathways, insulin sensitivity, and hormone regulation, providing insights into how physical activity can modify disease progression at a cellular level. This growing body of evidence will continue to refine exercise recommendations and further integrate physical activity into clinical practice.

### 5.4 Limitations of the current evidence base

It is critical to note that the mechanistic pathways proposed in this review are predominantly derived from preclinical animal models and pan-cancer cohorts. Direct randomized controlled trials evaluating exercise-induced vascular normalization and immune repolarization specifically within multimorbid, molecularly stratified EC populations are currently lacking. Furthermore, instances where exercise interventions have yielded modest or null effects highlight the heterogeneity of patient responses. Therefore, the “EC-comorbidity continuum” remains a hypothesis-generating framework requiring rigorous clinical validation before exercise can be prescribed as an established treatment sensitizer, rather than merely a tool for supportive care.

## 6 Conclusion

This review proposes repositioning physical activity from a general preventive measure to a targeted, investigational molecular modulator within a hypothetical endometrial cancer–comorbidity continuum. Based on a critical synthesis of current preclinical and indirect clinical data, we propose a testable dual-mechanism model: (1) Exercise-induced shear stress may promote eNOS phosphorylation and tumor vascular normalization, creating a “vascular bridge” hypothesized to enhance the delivery of chemotherapeutic agents and immunotherapies, particularly in patients with hypertension-driven endothelial dysfunction; and (2) Systemic metabolic reprogramming and reduced uremic/inflammatory burden may help repolarize Tumor-Associated Macrophages (TAMs) and partially reverse T-cell exhaustion, a mechanism potentially most relevant for sensitizing immunologically cold NSMP or p53-abnormal tumors to checkpoint blockade. We emphatically acknowledge that these conclusions are largely derived from preclinical models or extrapolation from other solid tumors, and rigorous validation in EC-specific clinical cohorts is an urgent prerequisite for translation. The high degree of biological heterogeneity in EC, as defined by its molecular subtypes, necessitates that future trials investigate exercise effects in a stratified manner. While personalized exercise oncology offers a promising, non-pharmacological strategy to address the formidable challenge of therapeutic resistance in multimorbid EC patients, its current clinical application remains primarily in the domains of supportive care and cardiovascular risk reduction. It should not yet be advocated as a proven treatment sensitizer, pending the outcome of targeted investigations.
